# Application study of the EQ-5D-5L in oncology: linking self-reported quality of life of patients with advanced or metastatic colorectal cancer to clinical data from a German tumor registry

**DOI:** 10.1186/s13561-020-00297-6

**Published:** 2020-12-12

**Authors:** Kathrin Borchert, Christian Jacob, Natalie Wetzel, Martina Jänicke, Egbert Eggers, Annette Sauer, Norbert Marschner, Julia Altevers, Thomas Mittendorf, Wolfgang Greiner

**Affiliations:** 1 Xcenda GmbH, Hannover, Germany; 2 iOMEDICO AG, Freiburg im Breisgau, Germany; 3 Kreiskrankenhaus Torgau, Torgau, Germany; 4 Medizinisches Versorgungszentrum für Blut- und Krebserkrankungen, Potsdam, Germany; 5 Praxis für interdisziplinäre Onkologie und Hämatologie, Freiburg im Breisgau, Germany; 6 Universität Bielefeld, Bielefeld, Germany

**Keywords:** Colorectal cancer, Tumor registry, EQ-5D-5L, Patient reported outcomes, Quality of life, Data linkage

## Abstract

**Background:**

The EQ-5D-5L questionnaire is used in oncology to generate health-related quality of life (HRQoL) weights and corresponding health states. The purpose was to explore the relationship between demographic and clinical characteristics and HRQoL among advanced or metastatic colorectal cancer (CRC) patients by linking clinical data of a German CRC registry to self-reported HRQoL measures from the EQ-5D-5L.

**Methods:**

The study sample included patients with advanced or metastatic CRC currently recruited in the German Tumor Registry Colorectal Cancer. The EQ-5D-5L was administered once to patients who were at the start or at later stages of palliative treatment. Data on comorbidities, disease-specific health states, symptoms, and treatment status were drawn from the registry. Multivariate regression analyses were performed to explore the impact of patient and disease characteristics on HRQoL.

**Results:**

In total, *n* = 433 questionnaires were included in the data analysis. Mean age of patients was 66.3 years and 61.2% were male. The mean EQ-5D-5L utility score was 0.82 and the mean EQ-5D-5L VAS score was 62.05. The regression analyses revealed that none of the demographic characteristics and few of the clinical characteristics, such as fatigue and pain, had a significant impact on the HRQoL.

**Conclusions:**

The study demonstrated a reduced HRQoL of patients with advanced or metastatic CRC when compared to the general population. The symptoms fatigue and pain negatively affected the HRQoL, whereas other characteristics such as age, gender, and comorbidities did not have a significant impact on HRQoL.

## Background

In Germany, colorectal cancer (CRC) is the second most frequent cancer among women and the third most frequent cancer among men. Approximately one out of eight cancers in Germany affects the bowel and about 6% of the population are diagnosed with colorectal carcinoma during their lifetime. Approximately 59,000 incident cases of CRC were forecasted in Germany for the year 2018 [1]. Patients with CRC suffer from a high psychological and physical burden of disease and have a reduced quality of life due to various problems in social functioning and disease-specific symptoms [2, 3].

The EuroQoL five-dimension questionnaire using a five-level scale (EQ-5D-5L) is widely used and well-accepted in oncology to measure health-related quality of life (HRQoL) [4]. However, for patients with advanced or metastatic CRC, there are no data available reporting the influence of certain comorbidities or health states on the HRQoL.

During the German benefit assessment (AMNOG) process, clinical trial data are evaluated regarding effectiveness and safety of new drugs compared to the standard of care, but also patient surveys are assessed for quality of life measures and health states. At the moment, quality adjusted life years (QALYs) assessed by the EQ-5D are not accepted in Germany for early benefit assessments, but the Institute for Quality and Efficiency in Health Care (IQWiG) is interested in conducting studies appreciating such data. The head of the Joint Federal Committee (G-BA) has suggested to further integrate HRQoL as a main outcome in the decision making process, also stating that missing data concerning patients’ QoL will have a negative impact on the overall assessment in the future [5].

Clinical registries routinely collecting clinical data present an opportunity to link HRQoL with data about disease status.

The aim of this cross-sectional application study was to explore the relationship between patient demographic, clinical characteristics and HRQoL measured with the EQ-5D-5L among patients with advanced or metastatic CRC in Germany by linking clinical data of the German colorectal cancer registry to self-reported HRQoL measures from the EQ-5D-5L. Results could serve as a basis for further research investigating if the EQ-5D-5L is able to generate (dis)utilities to be used in e.g. cost-effectiveness modelling among patients with advanced or metastatic CRC.

## Methods

### Data source

This cross-sectional application study derived clinical and patient-reported data from the Tumor Registry Colorectal Cancer (TKK) recruiting patients since September 2006 and run by the International Organisation of Medical Oncology (iOMEDICO). The TKK is a large, ongoing, prospective, national registry conducted by a multicenter network of 278 office- and hospital-based medical oncologists in Germany. The TKK was reviewed and approved by the Ethics Committee of the medical association of Baden-Württemberg, Germany. Data collection and methodology have been described previously [6].

At the time of study conduct, the TKK comprised data from about 6400 individuals. Inclusion criteria for the TKK are age ≥ 18 years, histologically confirmed CRC, and signed informed consent no longer than 4 weeks after the start of systemic neoadjuvant/adjuvant treatment for nonmetastatic or first-line treatment for metastatic/inoperable disease.

### Study population

The study population included histologically confirmed patients with advanced or metastatic CRC who had been recruited into the TKK since March 2014 and declared willingness to participate in patient-reported outcomes (PRO) surveys.

### Data collection

Potentially relevant patient and tumor characteristics (data of birth, gender, body mass index (BMI), comorbidities, primary tumor localization, operability of primary tumor), data on treatment (type of treatment per line of palliative line of treatment, antibody category, number of previous progressions, location of metastases) and treatment outcomes, and PROs were defined by literature review and by medical experts and extracted from the database as of the database cut May 2017.

iOMEDICO routinely sent out a PRO questionnaire to registered patients (at baseline, month 2, 4, 8, 12, 16, 20, and 24 after inclusion). This questionnaire contained the Quality of Life Questionnaire Core 30 version 3.0 of the European Organization for Research and Treatment of Cancer (EORTC QLQ-C30). For our application study, the EQ-5D-5L was added to the existing PRO questionnaire. The questionnaire was delivered once per patient by postal mail as part of the next round of questionnaire delivery between November 2016 and May 2017. Patients received a prepaid reply envelope and two reminders after two and 4 weeks.

### Applied questionnaires

The generic EQ-5D-5L questionnaire contains the five dimensions mobility, self-care, usual activity, pain/discomfort, and anxiety/depression. Each of these dimensions is to be evaluated by five levels ranging from no problems to extreme problems. In addition, the EQ-5D-5L includes the visual analogue scale (VAS), a continuous response scale ranging from 0 (worst possible health state) to 100 (best possible health state), to record patients’ self-rated health on the day of the interview [7, 8].

The EORTC QLQ-C30 is a questionnaire developed to assess the QoL of cancer patients. Version 3.0 includes 30 questions covering five function domains (physical, role, cognitive, emotional, and social), eight symptoms (fatigue, nausea/vomiting, pain, dyspnea, insomnia, appetite loss, constipation, and diarrhea), and financial impact on a four-point scale (1=“not at all” to 4 = “very much”) as well as overall health and overall QoL on a seven-point scale (1 = “very bad” to 7 = “excellent”). The questions refer to a one-week recall period called the “past week” [3, 9–11].

### Statistical methods

Utility scores from the EQ-5D-5L were calculated using a Germany-specific value set leading to scores from − 0.661 (worst possible health state) to 1 (best possible health state). VAS scores were recorded directly from the scale [7]. Scores from the EORTC QLQ-C30 were calculated following the scoring manual published by the EORTC leading to scores ranging from 0 to 100, with a higher score indicating a better level of functioning (functional scale) and a higher QoL (global health status/QoL scale), but a worse level of symptoms (symptom scale) [12]. Correlation between the three scores was tested using Pearson’s as well as Spearman’s correlation coefficient.

Tobit regression was applied to model the association between patient demographic and clinical characteristics and HRQoL. The tobit regression is designed to estimate linear relationships between variables when the dependent variable is either left- or right-censored. In this study, the dependent variable was HRQoL measured by the EQ-5D-5L. The score is bounded from above by 1.0 and it is common for a significant fraction of respondents to rate themselves in full health indicating that the score is unable to discriminate among high levels of health status. Therefore, we employed a tobit model with upper censoring at 1.0 in this study [13–16].

Two ordinary least squares (OLS) regression analyses on the EQ-5D-5L VAS score and the EORTC Global Health Status/QoL score were conducted and compared to the results from the tobit model.

The tobit regression of the EQ-5D-5L utility score and the OLS regression of the EQ-5D-5L VAS score were based on 377 patients because of missing data for individual variables. The OLS regression of the EORTC Global Health Status/QoL score was based on 366 patients due to missing data for individual variables.

All presented *p*-values are two-sided and considered to be statistically significant at < 0.05. All analyses were performed using the statistical software SAS version 9.2.

## Results

### Patient selection

**Fig. 1:**
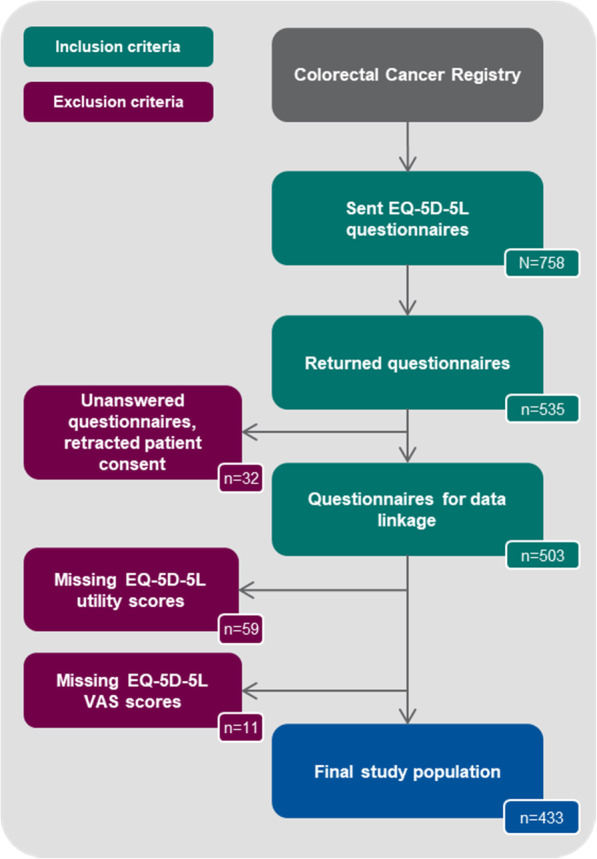
Flowchart of patient selection

In total, the PRO questionnaire including the EQ-5D-5L and EORTC QLQ-C30 was sent to *N* = 758 patients in the study period (between November 2016 and May 2017) of which *n* = 535 patients (70.6%) returned the questionnaire. After exclusion of unanswered questionnaires and questionnaires with retracted patient consents of *n* = 32 patients, data linkage of EQ-5D-5L data and clinical registry data was performed for *n* = 503 patients (66.4%). During data preparation, *n* = 59 patients were excluded due to missing EQ-5D-5L utility scores and *n* = 11 patients due to missing VAS scores. In total, *n* = 433 patients (57.1%) were included in the final data analysis (Fig. 1).

### Patient demographics and clinical characteristics

#### Demographics

At the time of PRO questioning, the patients were on average 66.3 years (±9.5 years) old and 61.2% of patients were male (*n* = 265). At the time of enrollment into the TKK registry, the mean BMI of these patients was 26.3 (±5.2).

#### Comorbidities

At the time of enrollment into the registry, 64.9% of the patients had at least one relevant comorbidity from the list: hypertension, adiposity, cerebrovascular disease, myocardial infarction, myocardial insufficiency, coronary heart disease, heart failure, peripheral artery occlusive disease, kidney disease, liver disease, diabetes mellitus, polyneuropathy, hemiplegia, peptic ulcer disease, urogenital disease, chronic gastric or bowel disease, thyroid disease, AIDS, anemia, connective tissue disease, dementia, tumor other than colorectal or lymphoma or leukemia, metastatic solid tumor other than colorectal. The most frequent comorbidity among the patients was hypertension (41.3%), followed by diabetes mellitus (14.6%) and thyroid disease (9.0%). Furthermore, coronary heart disease was prevalent in 8.3%, tumor other than colorectal or lymphoma or leukemia in 7.9%, chronic gastric or bowel disease in 6.7%, adiposity in 6.5%, and chronic pulmonary disease in 5.8%.

#### Tumor history and metastases

The primary tumor was located in the colon in 56.4% and in the rectum in 43.4% of cases. At the time of the initial diagnosis, 19.9% of the patients were diagnosed with an inoperable primary tumor. Tumor stage at primary diagnosis as classified by the Union for International Cancer Control (UICC) was I for 3.7% of the patients, II for 7.2%, III for 13.9%, and IV for 66.5%. The remaining patients (8.7%) had missing data.

About one-fifth of the patients (21.0%) had experienced at least one progression prior to questioning (16.6% with one progression and 4.4% with two or more progressions). At the time of last documentation before EQ-5D-5L questioning, 61.4% of patients had liver metastases, 23.8% had lung metastases, 14.6% had peritoneal metastases, and 3.0% had bone metastases. In 83.8% of patients, metastases were located at one or more localizations (51.2% one localization, 24.9% two localizations, 7.6% three or more localizations).

#### Disease or treatment-related symptoms

At the time of PRO questioning, the fatigue score calculated from the EORTC QLQ-C30 reached an average of 47.2 (±26.6), indicating a noticeable burden with regard to an achievable score range of 0 (lowest burden) to 100 (highest burden). The pain score was 26.6 (±30.4) and the nausea and vomiting score was 12.7 (±20.3) on average.

#### Palliative treatment status

At the time of PRO questioning, most patients were in first-line palliative therapy (55.2%), 23.3% were at break after first-line, 15.5% were in second-line or at break after second-line, and 5.1% in third-line or later. The most current chemotherapy at the time of questioning was based on irinotecan in 44.8% of the patients, oxaliplatin in 37.4%, fluoropyrimidine monotherapy in 10.4%, and both irinotecan and oxaliplatin in 4.9%. The remaining patients (2.5%) had missing data. Monoclonal antibodies that were given in addition to chemotherapy were Vascular Endothelial Growth Factor (VEGF)-inhibitors (49.7%) and Epidermal Growth Factor Receptor (EGFR)-inhibitors (26.1%), 22.6% of patients received no additional antibody treatment. 40.9% of the patients had tumors with Rat sarcoma (RAS) mutations and 38.1% presented with RAS wild-type tumors. The remaining 21.0% of the patients were either not tested or had missing data.

Comparing the demographic and clinical characteristics of the excluded patients (*n* = 70) with the included patients (*n* = 433) showed that the excluded patients tended to be older, to have more metastatic sites, and that their most current chemotherapy was composed of fluoropyrimidine monotherapy more often. These results indicate that among the already seriously ill group of patients with advanced or metastatic CRC, especially elderly and weakened patients and those with a particularly advanced CRC had to be excluded due to missing information in the EQ-5D-5L questionnaire (Table 1).

**Table 1:** Comparison of demographic and clinical characteristics of included and excluded CRC patients

	Included patients (***n*** = 433)	Excluded patients (***n*** = 70)	***p***-value*
**Mean age in years (SD)**	66.3 (9.5)	69.4 (9.9)	0.01^†^
**Male**	61.2%	68.6%	0.24
Mean BMI (SD)*	26.3 (5.2)	26.0 (3.8)	0.55
**Comorbidities at time of enrollment into registry**			
Hypertension	41.3%	45.7%	0.81
Diabetes mellitus with end-organ damage	0.7%	2.9%	0.27
Diabetes mellitus without end-organ damage	14.6%	18.6%	0.50
Thyroid disease	9.0%	4.3%	0.35
Coronary heart disease	8.3%	8.6%	1.00
Tumor other than colorectal or lymphoma or leukemia	7.9%	7.1%	1.00
Chronic gastric or bowel disease	6.7%	1.4%	0.18
Adiposity	6.5%	2.9%	0.44
Chronic pulmonary disease	5.8%	4.3%	0.90
Anemia	4.4%	4.3%	1.00
Heart failure	4.2%	7.1%	0.44
Kidney disease	3.5%	8.5%	0.15
Urogenital disease	3.2%	5.7%	0.07
Cerebrovascular disease	2.5%	1.4%	1.00
Myocardial infarction	2.5%	0.0%	0.47
Peripheral artery occlusive disease	2.1%	1.4%	1.00
Mild liver disease	1.4%	0.0%	1.00
Moderate or severe liver disease	0.7%	0.0%	1.00
Polyneuropathy	1.9%	1.4%	1.00
Peptic ulcer disease	0.5%	0.0%	1.00
Connective tissue disease	0.5%	0.0%	1.00
Metastatic solid tumor other than colorectal	0.5%	0.0%	1.00
Hemiplegia	0.2%	0.0%	1.00
AIDS	0.2%	0.0%	1.00
Myocardial insufficiency	0.0%	0.0%	1.00
Dementia	0.0%	1.4%	0.16
**Localization of primary tumor**			0.82
Colon	56.4%	58.6%	–
Rectum	43.4%	41.4%	–
**Inoperable primary tumor**	19.9%	24.3%	0.63
**Tumor stage (UICC) at primary diagnosis**			0.82
I	3.7%	4.3%	–
II	7.2%	5.7%	–
III	13.9%	11.4%	–
IV	66.5%	72.9%	–
**Progression prior to questioning**			0.27
0	75.3%	75.7%	–
1	16.6%	12.9%	–
2 or more	4.4%	7.1%	–
**Metastases**			
Liver	61.4%	58.6%	0.79
Lung	23.8%	21.4%	0.83
Peritoneal	14.6%	20.0%	0.49
Bone	3.0%	2.9%	0.93
**Number of metastatic localizations prior to questioning**			< 0.01^†^
0	10.9%	7.1%	–
1	51.2%	55.7%	–
2	24.9%	27.1%	–
3 or more	7.6%	5.7%	–
**Treatment line at time of questioning**			0.93
First-line palliative	55.2%	60.0%	–
Break after first-line	23.3%	20.0%	–
Second-line palliative or break after second-line	15.5%	14.3%	–
Third-line palliative or later	5.1%	5.7%	–
**Most current chemotherapy at time of questioning**			< 0.01^†^
Irinotecan-based	44.8%	44.3%	–
Oxaliplatin-based	37.4%	34.3%	–
Fluoropyrimidine monotherapy	10.4%	15.7%	–
Irinotecan plus oxaliplatin	4.9%	2.9%	–
**Additional antibody treatment**			0.68
None	22.6%	28.6%	–
VEGF	49.7%	42.9%	–
EGFR	26.1%	27.1%	–
**RAS mutation status**			0.74
Wild-type	38.1%	42.9%	–
Mutation	40.9%	40.0%	–

*Independent t-test was applied for continuous variables. Chi^2^-test or Fisher’s exact test was applied for categorical variables

^†^Statistically significant at alpha = 0.05

Percentages may not add up to 100% due to patients with missing data

### Patient-reported outcomes: EQ-5D-5L utility score, VAS score, and EORTC global health status/QoL score

**Fig. 2:**
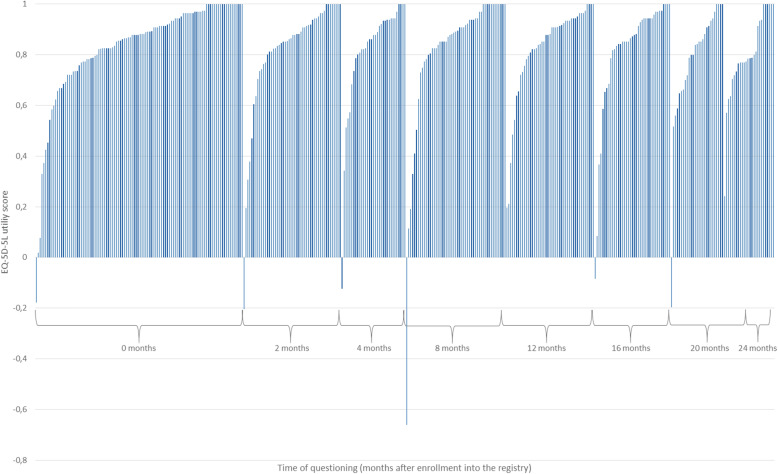
EQ-5D-5L utility scores by time of questioning

For most of the included patients (*n* = 332, 76.7%), the PRO questioning took place within the first 12 months after first-line palliative treatment initiation. The remaining patients (*n* = 101, 23.3%) received the questionnaire up until 24 months after first-line palliative treatment initiation. The mean time for all interviewed patients was 8.2 months (median 5.2 months) after first-line palliative treatment initiation. The distribution of EQ-5D-5L utility scores was similar in the different time of questioning groups with a slightly steeper slope for patients who received the PRO questionnaire after 20 or 24 months (Fig. 2).

Descriptive statistics of the EQ-5D-5L, the EQ-5D-5L VAS and the EORTC QLQ-C30 scores overall and stratified by time of questioning are shown in Table 2.

**Table 2:** Mean EQ-5D-5L utility scores, the EQ-5D-5L VAS scores, and the EORTC global health status/QoL scores

	EQ-5D-5L Utility Score		EQ-5D-5L VAS Score		EORTC Global Health Status/ QoL Score	
Time of questioning after enrollment	Mean	Standard Deviation (SD)	Mean	Standard Deviation (SD)	Mean	Standard Deviation (SD)
0 months	0.82	0.23	60.56	22.58	55.52	22.88
2 months	0.81	0.23	62.72	20.09	54.54	21.25
4 months	0.83	0.22	58.85	27.71	56.94	23.44
8 months	0.82	0.27	62.62	22.66	57.49	22.30
12 months	0.82	0.19	67.13	17.55	55.98	18.54
16 months	0.82	0.23	63.64	23.34	61.27	18.80
20 months	0.79	0.24	57.41	21.41	55.86	21.66
24 months	0.80	0.17	64.37	21.94	58.83	23.52
**Overall**	**0.82**	**0.23**	**62.05**	**22.23**	**56.66**	**21.66**

The included patients with advanced or metastatic CRC had an overall mean EQ-5D-5L utility score of 0.82 (±0.23). In comparison, the overall EQ-5D-5L VAS score and the overall EORTC global health status/QoL score were much lower with a mean of 62.05 (±22.23) and 56.66 (±21.66), respectively. Depending on the time of questioning after enrollment into the registry, the mean EQ-5D-5L score did not follow a linear trend but increased and decreased slightly between 0.83 (4 months) to 0.79 (20 months). The same findings were observed for the EQ-5D-5L VAS score with the highest score of 64.37 at 24 months and the lowest score of 57.41 at 20 months as well as for the mean EORTC global health status/QoL score with the highest score of 61.27 at 16 months and the lowest score of 54.54 at month two. All in all, the descriptive statistics indicated that the time of questioning did not seem to have an effect on the PRO scores in our patient sample which was confirmed in the regression analyses.

**Fig. 3:**
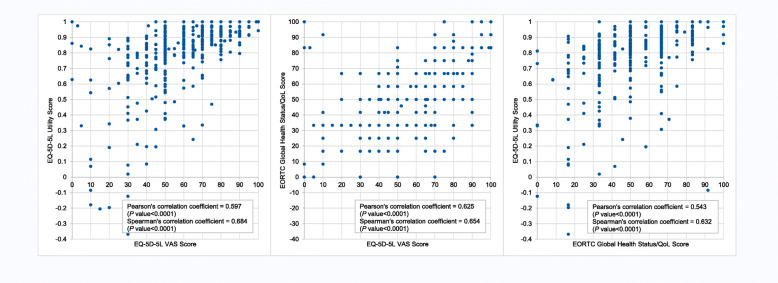
Correlation of EQ-5D-5L utility score, EQ-5D-5L VAS score, and EORTC global health status/QoL score

The correlation between the three scores was statistically significant (*P* < 0.0001) but also indicated a wider distribution of the EQ-5D-5L VAS scores and the EORTC global health status/QoL scores compared to the EQ-5D-5L utility scores (Fig. 3).

**Fig. 4:**
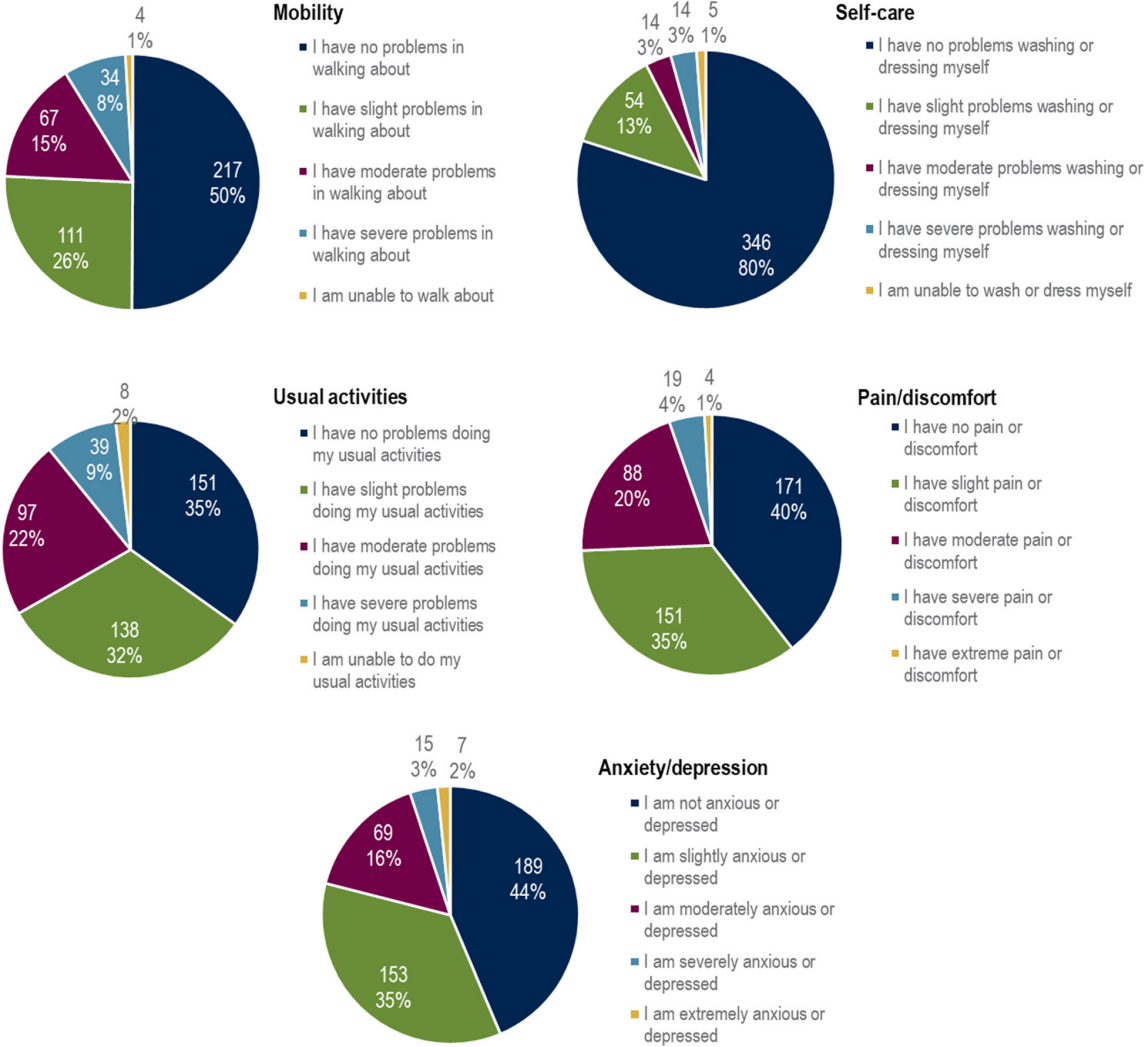
Level distribution in each EQ-5D-5L dimension

The detailed analysis of the five EQ-5D-5L dimensions showed that the majority of patients did not have any problems with self-care, whereas approximately two-thirds of the patients reported slight, moderate, or severe problems in performing usual activities or were even unable to perform them. Pain or discomfort was reported in 60.5% of the patients. 56.4% stated to be anxious or depressed and half of the patients had problems in walking about (Fig. 4). No problems or difficulties in any of the dimensions were reported in 16.9% of the patients.

### Association of patient characteristics and HRQoL: descriptive analysis

**Fig. 5:**
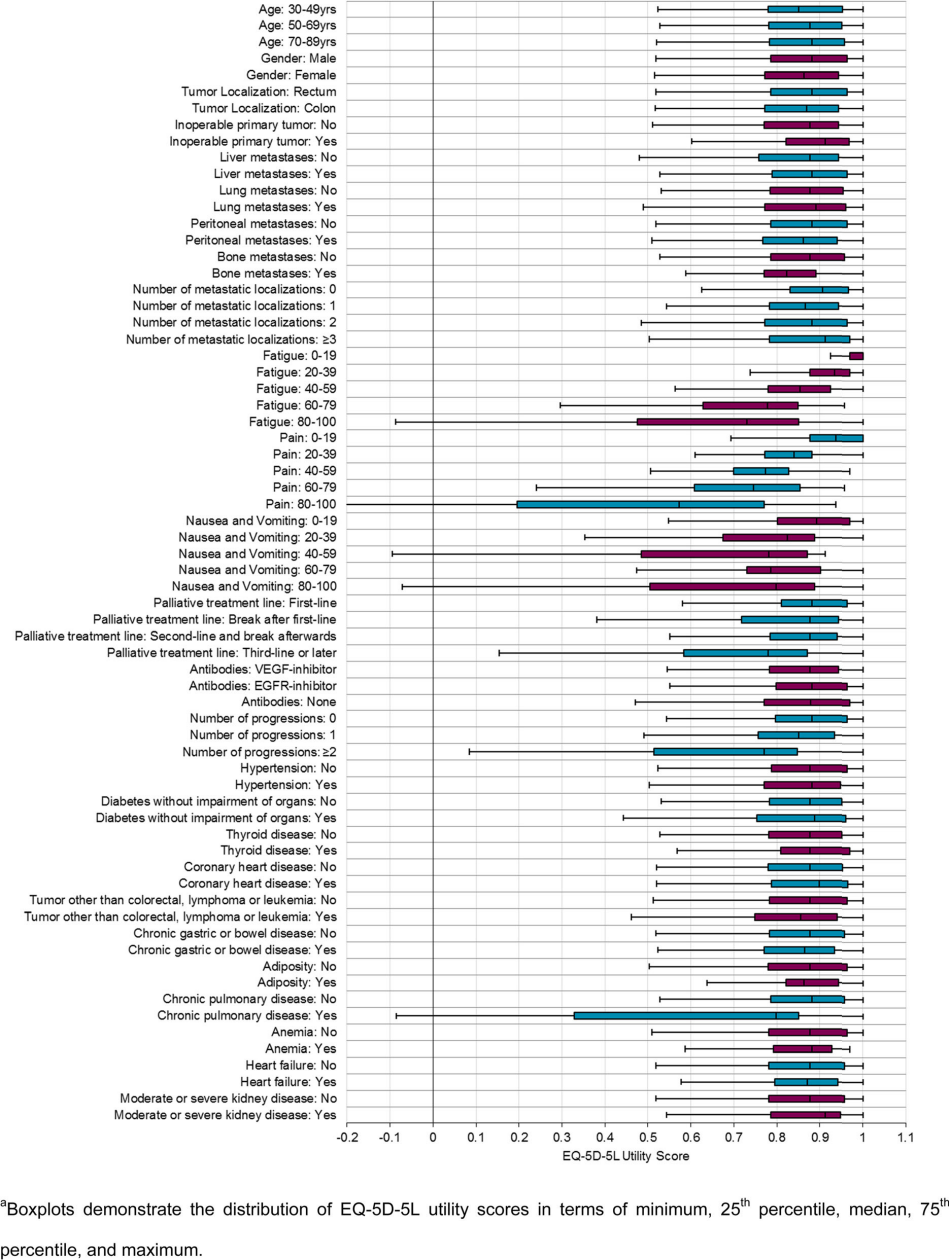
Distribution of EQ-5D-5L utility scores by patient characteristics^a^.

The boxplots in Fig. 5 illustrate the distribution of the EQ-5D-5L utility scores by patient characteristics in terms of minimum, 25th percentile, median, 75th percentile, and maximum. Regarding the demographic characteristics, the distribution was very similar showing no difference in terms of age groups and gender.

The categories of tumor history and metastases demonstrated a consistent distribution of HRQoL as well. Looking at the disease and treatment-related symptoms derived from the EORTC QLQ-C30, especially a high burden of fatigue and pain indicated a reduction in HRQoL. Patients undergoing third or later palliative treatment lines at the time of questioning had a remarkably lower HRQoL compared to patients in previous treatment lines. A decreased HRQoL was also shown for patients with two or more progressions in previous treatment lines than in patients with only one or no progression. For the noted comorbidities, patients with chronic pulmonary disease had the lowest HRQoL.

### Association of patient characteristics and HRQoL: regression analysis

Table 3 reports the results derived from the tobit and OLS regression analyses of the relationship between the EQ-5D-5L utility score, the EQ-5D-5L VAS score, the EORTC Global Health Status/QoL score and patients’ demographic and clinical characteristics, respectively.

**Table 3:** Tobit and linear regression analyses of the relationship between health state utilities and patients’ characteristics

	EQ-5D-5L Utility		EQ-5D-5L VAS		EORTC QoL	
	ß_**Tobit**_	SE	ß_**OLS**_	SE	ß_**OLS**_	SE
Intercept	1.092	0.101**	83.911	9.531**	74.268	8.717**
Time of PRO questioning after enrollment into the registry						
0 months	ref	ref	ref	ref	ref	ref
2 months	− 0.017	0.033	4.349	3.120	−0.270	2.847
4 months	−0.001	0.039	1.538	3.699	1.773	3.347
8 months	− 0.029	0.036	0.704	3.278	−4.164	2.961
12 months	− 0.027	0.037	8.225	3.497*	−0.533	3.216
16 months	0.004	0.040	6.400	3.714	6.568	3.384
20 months	−0.009	0.045	1.183	4.180	2.669	3.894
24 months	0.059	0.051	12.543	4.625**	7.240	4.268
Age	0.001	0.001	−0.079	0.102	0.070	0.092
Male	0.018	0.021	1.719	1.947	−1.069	1.791
BMI	0.001	0.002	0.200	0.207	0.127	0.197
Primary tumor localization						
Colon	ref	ref	ref	ref	ref	ref
Rectum	−0.006	0.021	0.189	1.916	0.201	1.750
Inoperable primary tumor	0.007	0.025	2.245	2.283	0.657	2.063
Number of progressions						
0	ref	ref	ref	ref	ref	ref
1	0.034	0.038	7.763	3.615*	−1.686	3.333
≥ 2	0.002	0.059	10.871	5.654	−5.621	5.151
Number of metastatic localizations						
0	ref	ref	ref	ref	ref	ref
1	−0.023	0.034	0.656	3.143	−1.168	2.894
2	−0.043	0.038	0.080	3.519	0.225	3.231
≥ 3	−0.053	0.049	0.522	4.539	1.627	4.131
Palliative treatment line						
First-line	ref	ref	ref	ref	ref	ref
Break after first-line	−0.047	0.029	−4.536	2.657	0.399	2.418
Second-line or later	−0.053	0.042	−11.939	4.000**	−1.377	3.725
Antibodies						
None	ref	ref	ref	ref	ref	ref
EGFR	0.012	0.029	−2.900	2.699	0.766	2.468
VEGF	0.022	0.025	−2.343	2.358	3.732	2.153
Comorbidities						
Adiposity	−0.010	0.043	1.318	4.063	0.577	3.707
Anemia	−0.055	0.045	−0.436	4.394	1.463	4.061
Chronic gastric or bowel disease	−0.015	0.039	−7.434	3.742*	−4.851	3.378
Coronary heart disease	0.022	0.039	−3.193	3.578	−4.569	3.235
Heart failure	0.029	0.051	−1.782	4.688	−2.827	4.245
Hypertension	−0.072	0.022**	−3.202	2.074	−2.377	1.904
Moderate or severe kidney disease	−0.026	0.056	−0.262	5.268	1.763	5.082
Diabetes mellitus^a^	0.031	0.029	1.637	2.746	2.521	2.502
Chronic pulmonary disease	−0.074	0.041	0.388	3.924	7.996	3.709**
Thyroid disease	−0.041	0.036	1.108	3.385	1.788	3.108
Tumor^b^	0.010	0.036	4.215	3.435	−4.265	3.134
Disease and treatment related symptoms (EORTC QLQ-C30 symptom scales)						
Nausea and vomiting	0.000	0.001	−0.011	0.051	−0.058	0.047
Fatigue	−0.004	0.000**	−0.384	0.044**	−0.472	0.040**
Pain	−0.003	0.000**	−0.170	0.036**	−0.121	0.033**
σ	0.177	0.007**	–	–	–	–
Pseudo-*R*^*2*^		0.035		–		–
Adjusted *R*^*2*^		–		0.386		0.485

*P < 0.05; **P < 0.01

^a^without organ impairment

^b^other than colorectal, lymphoma or leukemia

As opposed to OLS regression, coefficients from tobit regression analyses are not readily interpretable as effect sizes. Interpretation should consider whether the coefficient is negative or positive and whether it is statistically significant.

The results show that patients’ demographic characteristics did not affect HRQoL in any regression analysis. In addition, only few clinical characteristics had a statistically significant effect on HRQoL. In the tobit regression, hypertension, fatigue, and pain had a negative effect on the EQ-5D-5L utility score. In the OLS regression on the EQ-5D-5L VAS score, having completed the PRO questionnaire 12 or 24 months after enrollment into the registry and one prior progression had a positive effect on the EQ-5D-5L VAS score. Treatment in second-line or later, chronic gastric or bowel disease, fatigue, and pain, however, had a negative effect on the EQ-5D-5L VAS score. In the OLS regression of the EORTC Global Health Status/QoL score, chronic pulmonary diseases positively affected the EORTC Global Health Status/QoL score, but fatigue and pain had a negative effect.

In summary, all three regression analyses indicated that only few clinical characteristics influenced HRQoL. The symptoms fatigue and pain had a negative effect in all three regression analyses.

The marginal effects of the demographic or clinical characteristics from the tobit model were computed at the sample mean. Given the absence of significant differences in the impact of these characteristics over time, these marginal effects are based on the time invariant models reported in Table 3. They indicate that the statistically significant coefficients from the tobit regression only had a small effect on the EQ-5D-5L utility score: concomitant hypertension reduced the score by 0.052, each one-unit increase in the fatigue symptom scale by 0.003, and each one-unit increase in the pain symptom scale by 0.002.

## Discussion

This cross-sectional application study linking registry data to PRO data investigated the HRQoL among patients with advanced or metastatic CRC in Germany and its association with patient demographic and clinical characteristics. In total, *n* = 758 questionnaires were sent to patients, *n* = 535 were returned, and *n* = 433 were finally included in the data analysis. Mean age of patients was 66.3 years and 61.2% were male. The overall mean EQ-5D-5L utility score was 0.82, the mean EQ-5D-5L VAS score was 62.05, and the mean EORTC global health status/QoL score was 56.66. The tobit regression analysis revealed that hypertension, fatigue and pain had a significant negative impact on the HRQoL of advanced or metastatic CRC patients measured by the EQ-5D-5L utility score (*p* < 0.01). Concomitant hypertension reduced the HRQoL score by 0.052, each 1-unit increase in the fatigue symptom scale by 0.003 and in the pain symptom scale by 0.002.

Huang and colleagues [17] assessed the HRQoL of 300 newly diagnosed patients with CRC in China’s Heilongjiang province. On average, the included patients were younger (mean age 59 vs 66 years) and diagnosed at an earlier tumor stage (stage IV 12% vs 67%) than in our study. Nevertheless, they found a mean EQ-5D-5L utility score of 0.62 compared to 0.82 in our study. The proportion of patients reporting problems was similar for the dimensions pain/discomfort (60% vs 60%), anxiety/depression (59% vs 56%), and mobility (46% vs 50%), but differed for self-care (49% vs 20%) and usual activities (53% vs 65%).

The level distribution in the EQ-5D-5L dimension anxiety/depression showed that about 56% of CRC patients stated to be at least slightly to extremely anxious or depressed. It can be assumed that there is a relationship between physical and mental health as patients with CRC suffer physical burden of disease which might deteriorate their mental health status. Ohrnberger et al. [18] investigated the relationship between physical and mental health and possible mediators. They found that 8% of the total effect of physical health on mental health is mediated by physical health, with better physical health being correlated with higher physical activity which in turn has a positive impact on mental health [18].

Compared to the general population in Germany, problems in self-care (20.1% vs 6.4%) or pain and discomfort (60.5% vs 55.6%) were reported more frequently among patients with CRC. While 36.4% of the general German population did not report any problem or difficulty in any of the EQ-5D-5L dimensions [7], this proportion reached 16.9% among patients with CRC. Consequently, the patients with CRC analyzed in this study had a lower mean EQ-5D-5L utility score than the German general population (0.82 vs 0.92) [19]. However, this difference was smaller than expected and than observed for the EQ-5D-5L VAS score. Huber and colleagues [20] reported a mean EQ-5D-5L VAS score of 85.1 for the general population and Ludwig et al. from the EuroQoL group [7] found a mean score of 79.5 in their valuation study of the EQ-5D-5L. The mean score in the present study only reached 62.1 among patients with advanced or metastatic CRC. The relatively high and stable EQ-5D-5L utility scores may be explained by the generic nature of the EQ-5D-5L. The questionnaire may not detect delicate nuances that affect the HRQoL of cancer patients with an advanced or metastatic disease as it was not tailored for this specific group of patients. The VAS score might depict the current HRQoL state more accurately since it is not restricted to the five dimensions. Furthermore, non-random missing data have to be considered since patients with severely impaired HRQoL are more likely not to return the questionnaires.

Looking at the EORTC global health status/QoL score, the included patients with advanced or metastatic CRC presented with a lower mean score than rectal cancer patients without disease progression from the Munich Cancer Registry (56.7 vs 65.3–69.4) [3] which might indicate that HRQoL decreases with disease status. The symptom scores for fatigue, pain, and nausea and vomiting, on the other hand, were higher than in our study, indicating higher levels of symptomatology, and ranged between 64.8 and 68.6 for fatigue (vs 47.2 in our study), between 80.4 and 82.2 for pain (vs 26.6 in our study), and between 95.3 and 97.2 for nausea/vomiting (vs 12.7 in our study) [3]. The EORTC module for CRC (EORTC-QLQ-C29) is not routinely applied for patients in the TKK because answering 29 questions in addition to the EORTC-QLQ-C30 would be too burdensome for patients in palliative treatment situation.

The regression analyses revealed that none of the demographic and only few of the clinical characteristics had a significant impact on the HRQoL of patients with CRC. This applied to the tobit regression on the EQ-5D-5L utility score, the OLS regression on the EQ-5D-5L VAS score, and the OLS regression on the EORTC global health status/QoL score. In line with this, a review by Marventano and colleagues reported that gender was not a significant determinant of colorectal cancer patients’ HRQoL and that results on age were controversial [21]. They also found that symptoms induced by cancer or its treatment such as fatigue had a significant negative impact on HRQoL, which was confirmed in our study as both the fatigue and the pain score negatively affected the HRQoL in all three regressions. On the contrary, the review showed that heart disease had a significant role on overall HRQoL [21], which was not confirmed in our study. The study by Huang and colleagues [17] also assessed determinants of HRQoL of patients with CRC using tobit regression. The study found that low HRQoL was associated with more advanced stages of CRC, presence of a stoma, surgery only treatments, and low socioeconomic status. Treatment with surgery only or information on socioeconomic status and stoma was not available in our study. Data on stoma are not routinely collected in the TKK as the registry focuses on the real-world treatment with systemic therapies in Germany and the presence of a stoma does not have an impact on the treatment of a patient. Our findings did not indicate that more advanced stages of CRC were associated with a lower HRQoL. As pointed out by Huang et al. [17], the evidence for the association between HRQoL and the stage of CRC from the literature is contradictory.

An explanation for the few significant determinants of HRQoL might be that seriously ill patients have accepted their fate (coping) and recognize their disease, treatment, and comorbidities as part of their daily life. This behavior was also observed among cystic fibrosis patients [22]. Another explanation could be that the investigated comorbidities are under control and not relevant when compared to the symptoms associated with the cancer and its treatment. Furthermore, as mentioned above, data on patients with severely reduced HRQoL might be under-represented in our study because these patients were less likely to return the questionnaires.

There are two limitations that should be mentioned here in addition. First, specific patient characteristics such as comorbidities were only documented at start of palliative treatment. Consequently, changes in these characteristics until PRO questioning were not available and misclassification bias cannot be excluded. Second, confounding by unmeasured variables cannot be ruled out. Marventano and colleagues, for example, found in their review that depression, urinary disorder, a weak social network and a low income negatively affected HRQoL in patients with CRC [21], but these parameters were not collected in our study.

## Conclusion

This study linking clinical registry data to HRQoL data showed only a slight reduction in HRQoL of patients with advanced or metastatic CRC when compared to the general population or rectal cancer patients without disease progression. The regression analyses revealed that none of the included demographic and clinical characteristics had a significant impact on the HRQoL of patients with CRC except for the disease or treatment-related symptoms fatigue and pain. The relatively high and stable EQ-5D-5L utility scores could be caused by a high coping effect, by bias due to non-random missing data or by the generic character of the questionnaire not detecting delicate nuances. The VAS score seemed to depict the current HRQoL state more accurately since it is not restricted to five dimensions. However, this study demonstrates the feasible opportunity to collect additional data of interest in ongoing registries and that management of disease and treatment-related symptoms is of utmost importance to advanced and metastatic CRC patients.

## Data Availability

The datasets used and/or analyzed during the current study are available from the corresponding author on reasonable request. Restrictions apply to the availability of the data that supports the findings of this study, and so are not publicly available.

## Abbreviations

AMNOG: Act on the Reform of the Market for Medicinal Products (German benefit assessment)
BMI: Body Mass Index
CRC: Colorectal cancer
EGFR: Epidermal Growth Factor Receptor
EORTC QLQ-C30: Quality of Life Questionnaire Core 30 version 3.0 of the European Organization for Research and Treatment of Cancer
EQ-5D-5L: EuroQoL five-dimension questionnaire using a five-point scale
G-BA: Head of the Joint Federal Committee
HRQoL: Health related quality of life
iOMEDICO: International Organisation of Medical Oncology
IQWiG: Institute for Quality and Efficiency in Health Care
OLS: Ordinary least squares
PRO: Patient-reported outcomes
QALYs: Quality adjusted life years
RAS: Rat sarcoma
SD: Standard Deviation
TKK: Tumor Registry Colorectal Cancer
UICC: Union for International Cancer Control
VAS: Visual analogue scale
VEGF: Vascular Endothelial Growth Factor
